# Simulation study of reducing reflection losses in all-perovskite tandem solar cells through dual serrated structure

**DOI:** 10.1007/s12200-025-00153-7

**Published:** 2025-04-22

**Authors:** Wenjiang Ye, Aoyue Chen, Ping Fu, Jiang Tang, Chao Chen

**Affiliations:** 1 Wuhan National Laboratory for Optoelectronics (WNLO) and School of Optical and Electronic Information (SOEI), Huazhong University of Science and Technology, Wuhan, 430074 China; 2 China-EU Institute for Clean and Renewable Energy, Huazhong University of Science and Technology, Wuhan, 430074 China; 3 State Key Laboratory of Photoelectric Conversion and Utilization of Solar Energy, Dalian Institute of Chemical Physics, Chinese Academy of Sciences, Dalian, 116023 China; 4 Optics Valley Laboratory, Wuhan, 430074 China; 5 Hubei Optical Fundamental Research Center, Wuhan, 430074 China

**Keywords:** Reflection loss, Tandem solar cells, Perovskite, Microstructure

## Abstract

The power conversion efficiency of all-perovskite tandem solar cells is predominantly constrained by optical absorption losses, especially reflection losses. In this simulation study, we propose the optimization of a dual-interface serrated microstructure to mitigate these optical reflection losses in all-perovskite tandem solar cells. By adjusting the geometry of the periodic serrated structures at both the front interface and the back electrode, we enhance light absorption in the wide-bandgap perovskite layer and promote light scattering in the narrow-bandgap perovskite layer. The structural modification reduces the reflection-induced photocurrent density loss from 4.47 to 3.65 mA cm^−2^. It is expected to boost the efficiency of all-perovskite tandem solar cells to approximately 31.13%, representing a 3.41% increase. The dual-interface optimization effectively suppresses reflection losses and improves the overall photocurrent of all-perovskite tandem solar cells. These results offer a promising strategy for minimizing optical losses and enhancing device performance in all-perovskite tandem solar cells.

**Graphical Abstract:**

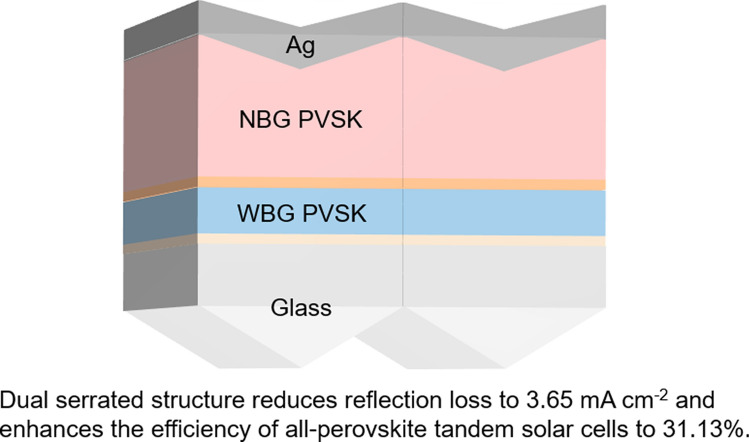

**Supplementary Information:**

The online version contains supplementary material available at 10.1007/s12200-025-00153-7.

## Introduction

All-perovskite tandem solar cells (TSCs) have achieved a power conversion efficiency (PCE) of 30.1% [1], yet significant optical losses prevent them from reaching the theoretical maximum efficiency of 45% [2, 3]. Among these losses, reflection-induced energy dissipation is particularly severe. Research indicates that, for a specific structure of all-perovskite TSCs, optical losses account for approximately 31.15% of the total energy loss, with reflection losses contributing as much as 19.64% [2]. These reflections not only reduce the photon flux reaching the absorption layers but also create a critical bottleneck in achieving the theoretical efficiency limit.

To mitigate reflection losses, Huang et al. increased the photon path length by embedding micrometer-sized resin particles in narrow-bandgap (NBG) perovskite, resulting in an absorption enhancement of ~ 10% in the infrared region for the NBG perovskite layer [4]. Tan and colleagues optimized the absorption of sunlight in both the wide-bandgap (WBG) and NBG perovskite layers by adjusting their respective thicknesses, which reduced reflection losses and enabled the realization of all-perovskite TSCs with a photocurrent of 15.5 mA cm^−2^ [5]. Fang and collaborators minimized reflection losses across the entire spectrum by approximately 5% by coating glass substrates with highly distributed nanoplates of fluorine-doped tin oxide [6]. These studies have significantly reduced light reflection losses in all-perovskite TSCs through techniques such as light-scattering structures, thickness optimization, and material enhancements [7–10]. Despite these advancements, the efficiency still falls substantially short of the theoretical limit for all-perovskite TSCs.

In this work, we analyzed the primary optical losses in all-perovskite TSCs through systematic simulation analyses. By precisely regulating the optical characteristics of the periodic front serrated structure to optimize the light incident path, we successfully reduced the reflection intensity near 350 nm by approximately 10%. Furthermore, the optimized periodic back serrated structure significantly enhanced light scattering and diffraction effects, reducing the reflection intensity near 950 nm by 5%. The structural modification reduced the reflection-induced photocurrent density loss from 4.47 to 3.65 mA cm^−2^. It is expected to boost the efficiency of all-perovskite TSCs to approximately 31.13%, representing a 3.41% increase. The enhanced light absorption efficiency now approaches the theoretical limit. This study provides both a theoretical foundation and innovative design strategies for advancing the performance of all-perovskite TSCs.

## Simulation methodology

In this simulation, we primarily established an optical model to optimize the optical absorption performance of the device to demonstrate the performance of all-perovskite TSCs [11–14].

Based on a typical all-perovskite TSC structure, we employed COMSOL Multiphysics to simulate the optical response, utilizing Floquet–Bloch boundaries and perfectly matched layers. Specifically, the optical properties, such as light absorption, reflection, and transmission, were determined by solving Maxwell’s equations:

1 $$\nabla \times \vec{E} = - \frac{{\partial \vec{B}}}{\partial t},$$

2 $$\nabla \times \vec{H} = - \frac{{\partial \vec{D}}}{\partial t} + \vec{J},$$

3 $$\nabla \cdot \vec{D} = \rho ,$$

4 $$\nabla \cdot \vec{B} = 0,$$

5 $$\nabla \times \left( {\nabla \times E} \right) = { }k_{0}^{2} \varepsilon_{{\text{c}}} E,$$

6 $$G\left( {x,y,z} \right) = \smallint g\left( {x,y,z,\lambda } \right){\text{d}}\lambda ,$$

where $E$ is the electromagnetic field, *B* is the magnetic flux density, *D* is the electric displacement vector, *J* is the conduction current density, ${k}_{0}$ is the wave vector in free space, ${\varepsilon }_{\text{c}}$ is the frequency-dependent complex permittivity, *G* (*x*, *y*, *z*) is the spatial distribution of generated carrier, and *g*(*x*, *y*, *z*, *λ*) is the photogeneration rate of the carriers at a specific wavelength *λ* in position (*x*, *y*, *z*).

Then we calculate the photocurrent density absorbed by the two absorption layers based on the following equations:

7 $$J_{{{\text{ph}}\_{\text{WBG}}}} = \int {\int {\int {\int_{{{\text{WBG}}}} {g\left( {x,y,z,{\lambda }} \right){\text{d}}x{\text{d}}y{\text{d}}z{\text{d}}\lambda ,} } } }$$

8 $$J_{{{\text{ph}}\_{\text{NBG}}}} = \int {\int {\int {\int_{{{\text{NBG}}}} {g\left( {x,y,z,{\lambda }} \right){\text{d}}x{\text{d}}y{\text{d}}z{\text{d}}\lambda .} } } }$$

## Results and discussion

### Reflection losses in all-perovskite TSCs

**Fig. 1:**
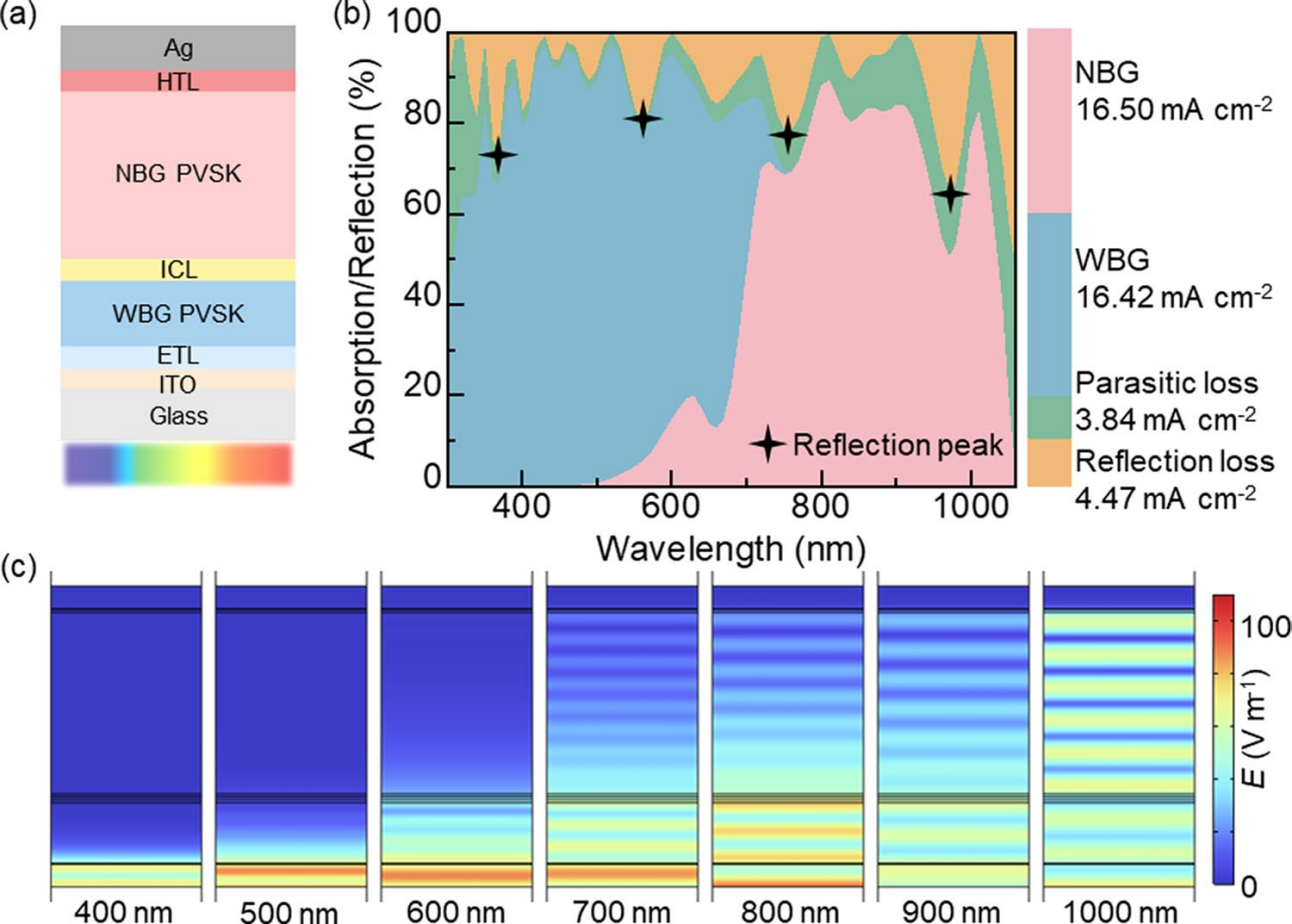
**a** Illustration of a typical all-perovskite TSC structure. **b** Absorption characteristics and photocurrent density distribution across WBG subcell, NBG subcell, parasitic loss and reflection loss of the all-perovskite TSCs. **c** Electric field intensity distribution at different wavelengths of the flat structure

First, we modeled the typical structure of the ultra-high-efficiency all-perovskite TSCs reported to date (Fig. 1a), to systematically analyze their optical performance [3]. Through precise optical simulations, we quantified the light absorption distribution across different parts (Fig. 1b) [9]. The photocurrent densities of the WBG and NBG perovskite layers are 16.42 mA cm^−2^ and 16.50 mA cm^−2^, respectively. This indicates that balanced photocurrent densities are generated in both the WBG and NBG subcells, consistent with the literature reported (Table S2) [3]. The bandgaps of the WBG and NBG perovskite layers are 1.8 eV and 1.25 eV, respectively. The complex refractive indices of the materials of each layer are shown in Fig. S4. Subsequently, through thickness scan analysis, we found that the device performance reaches its optimal state when the thickness of the WBG layer is 400 nm and that of the NBG layer is 1200 nm (Fig. S1, Table S1). Reflection loss accounts for 10.84% (4.47 mA cm^−2^) of the total photocurrent density within the spectral region (Fig. S2). This indicates that reflection loss significantly impacts overall device performance. Furthermore, spectral analysis reveals prominent reflection peaks with intensities exceeding 20% at multiple wavelengths, including 350 nm, 550 nm, 750 nm, and 950 nm. These peaks can be attributed to the refractive index mismatch at the front interface, causing some incident light to be reflected rather than effectively coupled into the perovskite absorption layer [9]. Therefore, reducing reflection loss is crucial for improving the efficiency of all-perovskite TSCs.

### Reducing reflection losses through front serrated structure

**Fig. 2:**
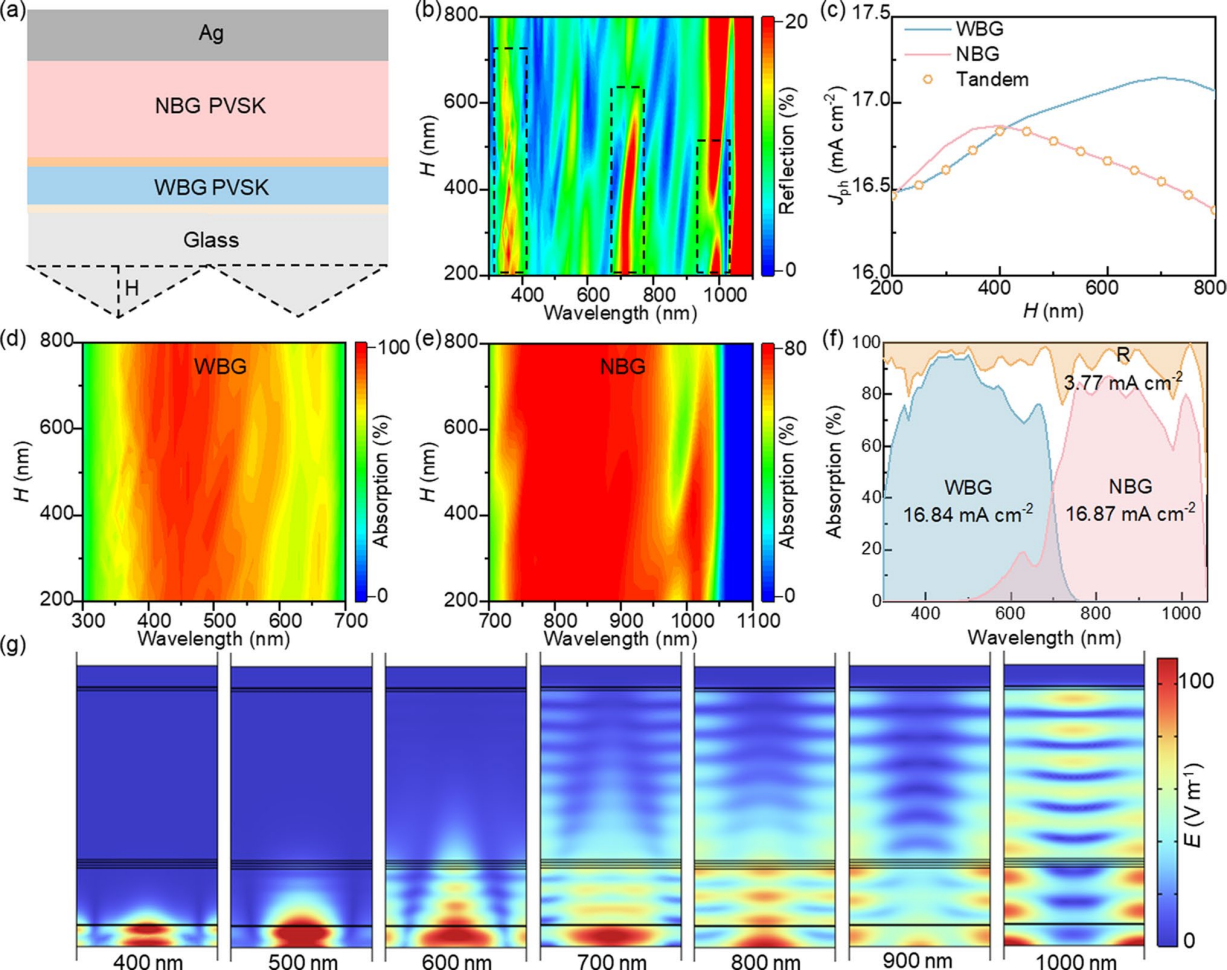
**a** Schematic illustration of the periodic front serrated structure. **b** Reflection at different wavelengths for various heights of the front serrated structure. **c** *J*_ph_ of WBG and NBG subcells for various heights of the front serrated structure. **d** Absorption at different wavelengths in the WBG perovskite layer for various heights of the front serrated structure. **e** Absorption at different wavelengths in the NBG perovskite absorption layer for various heights of the front serrated structure. **f** Absorption of WBG and NBG subcells and total absorption (1-R) of all-perovskite TSCs optimized by the front serrated structure. **g** Electric field intensity distribution at different wavelengths when the height of the front serrated structure is 400 nm

The serrated structure has been proven to control the light path and is commonly used to reduce reflection losses in solar cells [15–18]. Here, we designed a periodic serrated structure with a fixed periodicity of 1 μm on the front interface and varied the height (*H*) of the front serrated structure, which significantly affects light incidence, as shown in Fig. 2a.

As shown in Fig. 2b, the variation in reflection with *H* shows that the reflection intensity decreases as *H* increases in the range of 300–700 nm. In the range of 700–1000 nm, the reflection intensity initially decreases and then increases as *H* increases, reaching a minimum of around 10% when *H* = 300 nm. Reflection directly affects the absorption of the subcells, so we analyzed how the absorption of the WBG and NBG subcells changes with *H*. The absorption intensity of the WBG subcell around 550 nm first increases and then decreases as *H* increases, in Fig. 2d. For the NBG subcell, the absorption intensity at around 1000 nm increases with *H*, then decreases, reaching a maximum of 80% when *H* = 400 nm, as shown in Fig. 2e.

Furthermore, the absorption of the subcells determines the photocurrent density they generate. We calculated the photocurrent densities of the WBG and NBG subcells at different heights of the front serrated structure. The photocurrent density of the WBG subcell increases with *H*, reaches a maximum of 17.15 mA cm^−2^ when *H* = 700 nm, and then decreases. The photocurrent density of the NBG subcell increases with *H*, reaches a maximum of 16.87 mA cm^−2^ when *H* = 400 nm, and then decreases. The photocurrent density of the all-perovskite TSCs is limited by the NBG subcell, which has the lower current. It increases and then decreases, reaching a maximum of 16.87 mA cm^−2^ when *H* = 400 nm (Fig. 2c). The trend in photocurrent aligns with the changes observed in the spectra.

To further analyze the effect of the front serrated structure on the internal light field distribution of the all-perovskite TSCs, we calculated the electric field distribution at different wavelengths inside the device when *H* = 400 nm (Fig. 2g). In the absorption range of the WBG subcell (400–700 nm), the front serrated structure acts like a micro-lens, focusing and significantly enhancing the electric field intensity at the front interface [15]. The maximum electric field intensity reaches 100 V m^−1^, which is 4 times higher than in the flat structure (Fig. 1c). At a wavelength of 1000 nm, the front serrated structure disrupts the waveguide modes in the flat structure, significantly increasing the maximum electric field intensity in the NBG perovskite layer to 70 V m^−1^, 1.5 times increase compared to the flat structure. These phenomena show that the optimized front serrated structure (*H* = 400 nm) effectively controls the light propagation path, increasing the light transmission length within the perovskite absorption layer, especially in the WBG perovskite layer, thus optimizing the light trapping and absorption process.

With the optimization of the front serrated structure (*H* = 400 nm), the reflection intensity in the range of 300–700 nm decreases by about 5%, and the reflection intensity in the range of 700–1000 nm decreases by about 10% (Fig. 2b). The reflection loss current density drops from 4.47 to 3.77 mA cm^−2^, a 15.66% reduction compared to the flat structure. The photocurrent density of the all-perovskite TSCs reaches a peak of 16.84 mA cm^−2^, which is a 2.56% increase over the flat structure (Fig. 2f). However, there is still about 20% reflection loss at around 950 nm, which limits the photocurrent of the all-perovskite TSCs due to the NBG subcell. Therefore, it is necessary to further optimize the reflection loss in the long-wavelength region.

### Reducing reflection losses through back serrated structure

**Fig. 3:**
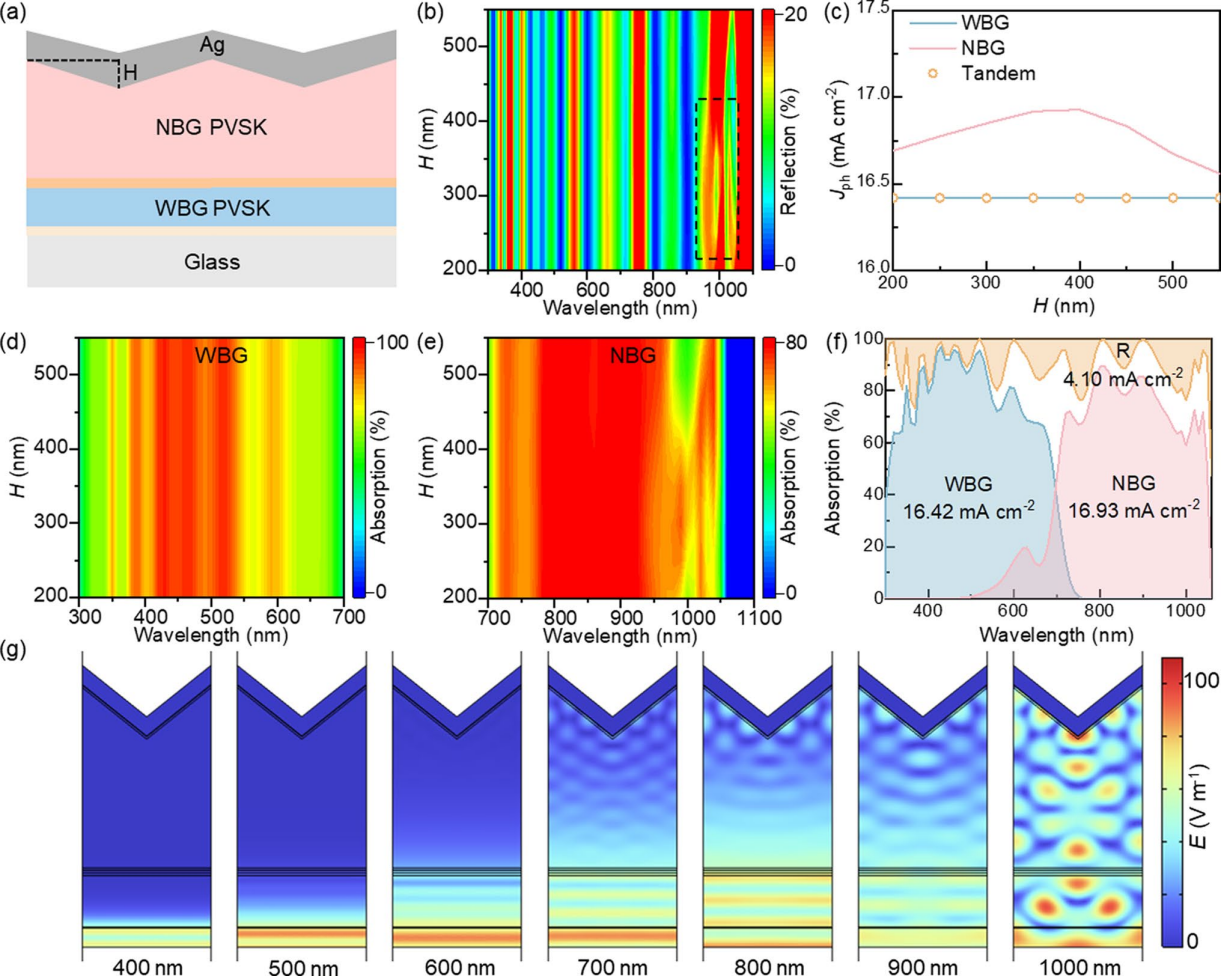
**a** Schematic illustration of the periodic back serrated structure. **b** Reflection at different wavelengths for various heights of the back serrated structure. **c** *J*_ph_ of WBG and NBG subcells for various heights of the back serrated structure. **d** Absorption at different wavelengths in the WBG perovskite layer for various heights of the back serrated structure. **e** Absorption at different wavelengths in the NBG perovskite absorption layer for various heights of the back serrated structure. **f** Absorption of WBG and NBG subcells and total absorption (1-R) of all-perovskite TSCs optimized by the back serrated structure. **g** Electric field intensity distribution at different wavelengths when the height of the back serrated structure is 400 nm

To reduce reflection losses in the long-wavelength range, we designed the same periodic serrated structure on the back electrode, involving nanoimprinting the perovskite layer followed by metal cathode deposition. Then we varied the height (*H*) of the back serrated structure (Fig. 3a) [15].

The reflection intensity changes with *H* indicate that the reflection intensity remains nearly constant as *H* varies in the range of 300–700 nm. At around 1000 nm, the reflection intensity decreases and then increases as *H* increases, reaching a minimum of about 15% when *H* = 400 nm (Fig. 3b). Similarly, we analyzed the changes in absorption of the WBG and NBG subcells with varying *H*. The absorption of the WBG subcell remains constant as *H* increases (Fig. 3d). The absorption of the NBG subcell increases and then decreases with increasing *H*, showing multiple absorption peaks around 1000 nm when *H* = 400 nm (Fig. 3e).

Furthermore, we calculated the photocurrent density of the WBG and NBG subcells at different values of *H*. The photocurrent density of the WBG subcell remains constant at 16.42 mA cm^−2^ as *H* increases. The photocurrent density of the NBG subcell initially increases and then decreases with increasing *H*, reaching a maximum of 16.93 mA cm^−2^ when *H* = 400 nm. The photocurrent density of the all-perovskite TSCs is limited by the lower current of the WBG subcell, remaining at 16.42 mA cm^−2^ (Fig. 3c). This trend in photocurrent is consistent with the changes observed in the spectral.

Similarly, we calculated the electric field distribution inside the device at different wavelengths when *H* = 400 nm (Fig. 3g). In the absorption range of the WBG subcell (400–700 nm), the back serrated structure has minimal impact on the electric field strength. However, in the long-wavelength absorption range of the NBG subcell (700–1000 nm), the optimized back serrated structure significantly promotes photon cycling within the NBG perovskite layer. This increases the maximum electric field strength to 80 V m^−1^, which is nearly 1.7 times higher than the uniformly decaying electric field strength observed in the flat structure (Fig. 1c). Additionally, infrared light excites surface plasmon polariton (SPP) coupling at the serrated tips of the Ag/NBG interface, enhancing near-field effects and improving the NBG perovskite layer’s ability to absorb long-wavelength light.

Through the optimization of the back serrated structure (*H* = 400 nm), the reflection intensity decreases by about 5% in the range of 700–1000 nm (Fig. 3b), and the reflection loss photocurrent density drops from 4.47 to 4.10 mA cm^−2^, representing an 8.28% reduction compared to the flat structure. The photocurrent density of the NBG subcell increases to 16.93 mA cm^−2^, a 2.61% improvement over the flat structure. However, the photocurrent density of the all-perovskite TSCs remains limited by the WBG subcell, staying at 16.42 mA cm^−2^ (Fig. 3f). To further enhance the photocurrent density of all-perovskite TSCs, it is crucial to optimize the reflection loss in combination with the front serrated structure.

### Reducing reflection losses through dual serrated structure

**Fig. 4:**
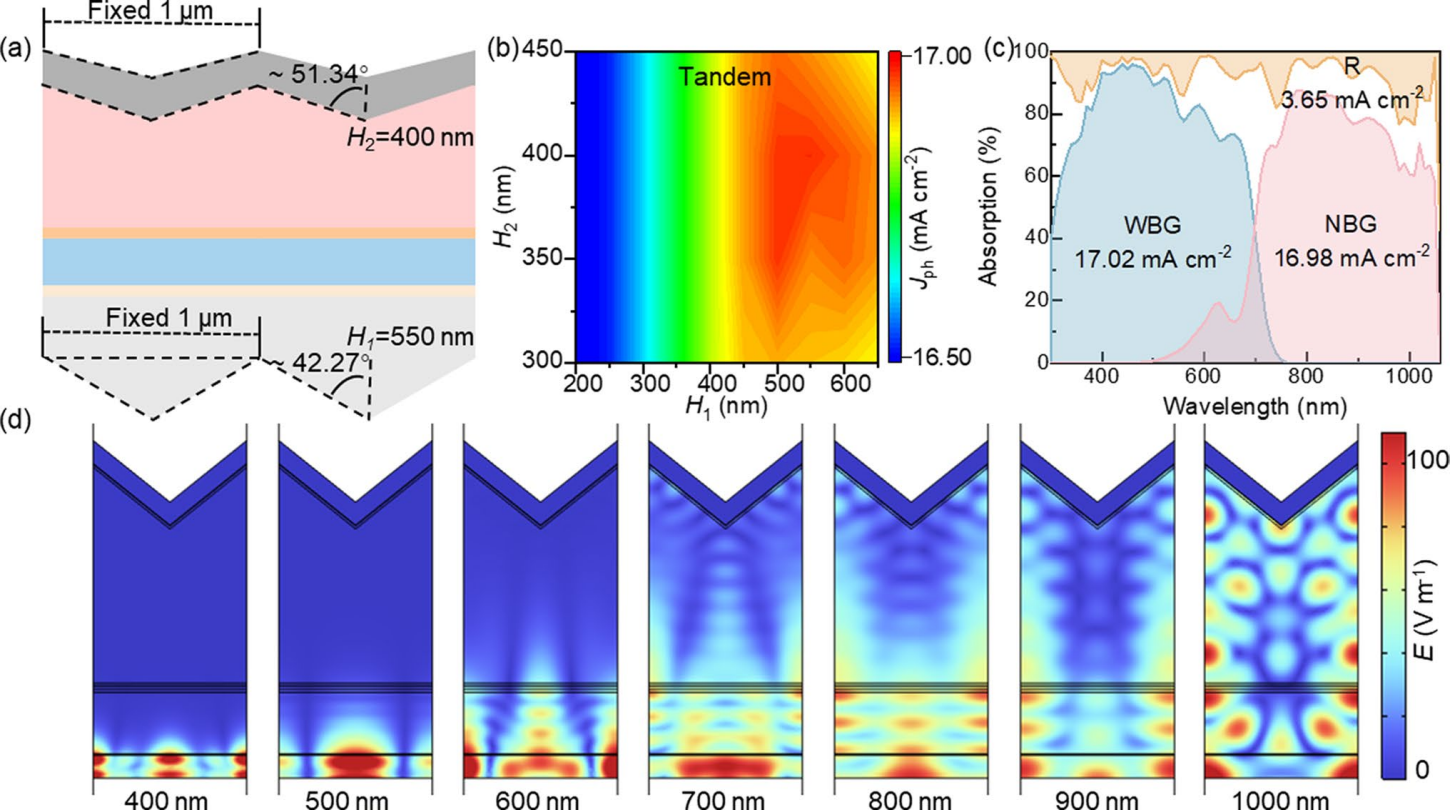
**a** Schematic illustration of the periodic dual serrated structure. **b** *J*_ph_ of all-perovskite TSCs with the height *H*_1_ of the front serrated structure and the height *H*_2_ of the back serrated structure. **c** Absorption of WBG and NBG subcells and total absorption (1-R) of all-perovskite TSCs optimized by the dual serrated structure. **d** Electric field intensity distribution at different wavelengths when the height of the front serrated structure is 550 nm and the height of the back serrated structure is 400 nm

On one hand, when the height of the front serrated structure exceeds 400 nm, the reflection intensity in the range of 300–700 nm continues to decrease as *H* increases. However, the reflection intensity around 1000 nm increases, preventing further enhancement of the tandem device’s performance (Fig. 2b). On the other hand, the optimized back serrated structure reduces reflection in the NBG subcell without affecting the WBG subcell (Fig. 3b). Combining these two aspects, we designed a periodic dual serrated structure (Fig. 4a), with the front serrated structure height of *H*_1_ and the back serrated structure height of *H*_2_. We explored the synergistic optimization of the front (*H*_1_) and back (*H*_2_) serrated heights and their impact on the photocurrent density of all-perovskite TSCs [10, 19, 20]. Through optimization, the dual serrated structure with front and back serrated heights of 550 nm (*H*_1_) and 400 nm (*H*_2_) reduces reflection and enhances absorption (Fig. S3), achieving a maximum photocurrent density of 16.98 mA cm^−2^ (Fig. 4b).

Similarly, we calculated the electric field distribution inside the device at different wavelengths for the optimized dual serrated structure (*H*_1_ = 550 nm, *H*_2_ = 400 nm) (Fig. 4d). In the absorption range of the WBG subcell (400–700 nm), the device exhibited significant optical focusing effects, greatly enhancing the electric field strength. The maximum electric field strength reached 100 V m^−1^, which is 4 times higher than the electric field strength in the flat structure, fully demonstrating the optical effect of the front serrated structure. At a wavelength of 1000 nm, the dual serrated structure improved light scattering and absorption in the long-wavelength region [21, 22]. This increased the maximum electric field strength in the NBG perovskite layer to 100 V m^−1^, which is twice as high as that in the flat structure.

Overall, the front serrated structure design effectively enhanced light capture and propagation, improving the absorption efficiency in absorption range of the WBG subcell (300–700 nm), while the serrated structure on the back electrode enhanced light scattering and trapping effects, significantly increasing absorption range of the NBG subcell (700–1000 nm) [23, 24]. Through the optimization of the periodic dual serrated structure (*H*_1_ = 550 nm, *H*_2_ = 400 nm), the reflection loss photocurrent density decreases from 4.47 to 3.65 mA cm^−2^, representing an 18.34% reduction compared to the flat structure. It is expected to boost the efficiency of all-perovskite TSCs to approximately 31.13%, representing a 3.41% increase (Fig. 4c).

## Conclusion

In conclusion, we designed a dual-interface serrated microstructure optimization for all-perovskite TSCs to address the challenges posed by optical reflection losses. The proposed design reduces reflection-induced photocurrent density loss from 4.47 to 3.65 mA cm^−2^ (an 18.34% reduction) while synergistically enhancing WBG layer absorption and NBG layer light trapping. It is expected to boost the efficiency of all-perovskite TSCs to approximately 31.13%, representing a 3.41% increase. Our study demonstrates that the synergistic optimization of both the front interface and back electrode optical structures is an effective strategy for improving the efficiency of all-perovskite TSCs. Future research will focus on further minimizing residual reflection losses while ensuring current matching between the WBG and NBG subcells to fully realize the potential of all-perovskite TSCs.

## Supplementary Information

Supplementary Material 1.

## Data Availability

The data that support the findings of this study are available from the corresponding author, upon reasonable request.
