# Supplementary material for: Simulation study of reducing reflection losses in all-perovskite tandem solar cells through dual serrated structure
Source: Front Optoelectron. 2025 Apr 22;18(1):9. doi: 10.1007/s12200-025-00153-7 (PMC12014966; doi:10.1007/s12200-025-00153-7)
Supplement: Supplementary file 1 — Supplementary Material 1. [file 12200_2025_153_MOESM1_ESM.pdf]

Supporting Information for:

Simulation study of reducing reflection losses in all-perovskite tandem solar cells through dual serrated structure

Wenjiang Ye [1,2†], Aoyue Chen [1†], Ping Fu [3], Jiang Tang[1,2,4,5], Chao Chen[1,2,4,5*]

[1] Wuhan National Laboratory for Optoelectronics (WNLO) and School of Optical and Electronic Information (SOEI), Huazhong University of Science and Technology; Wuhan, 430074, China.

[2] China-EU Institute for Clean and Renewable Energy, Huazhong University of Science and Technology; Wuhan 430074, China.

[3] State Key Laboratory of Photoelectric Conversion and Utilization of Solar Energy, Dalian Institute of Chemical Physics, Chinese Academy of Sciences; Dalian, 116023, China.

[4] Optics Valley Laboratory; Wuhan, 430074, China.

[5] Hubei Optical Fundamental Research Center; Wuhan, 430074, China.

[†] These authors contributed equally to this work.

* Corresponding author.

Email address: cchen@hust.edu.cn

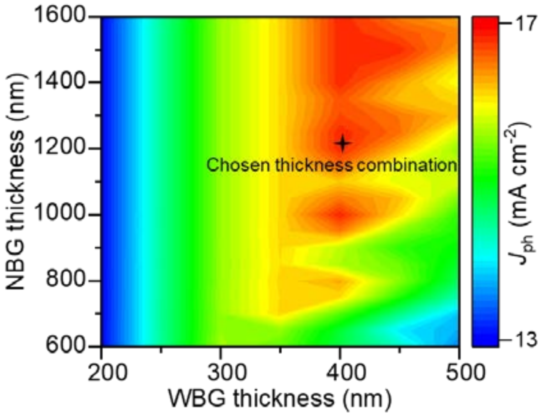

Fig. S1 J_{ph} of all-perovskite TSCs as a function of the thickness of WBG and NBG perovskite layers.

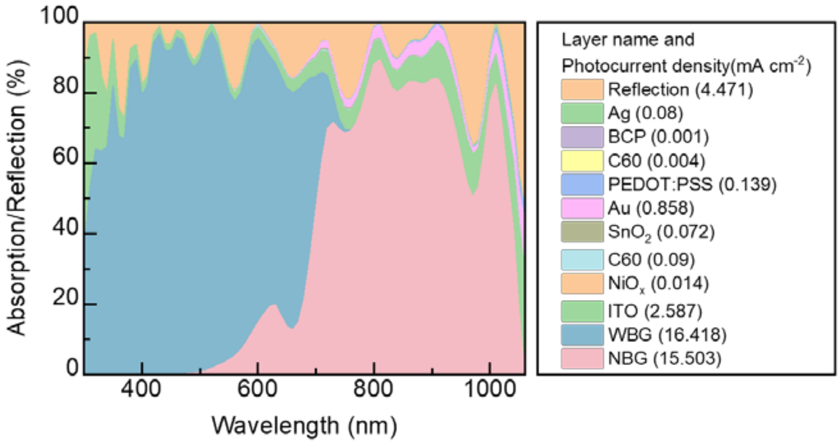

Fig. S2 Optical analysis of the all-perovskite TSCs of each layer and reflection loss.

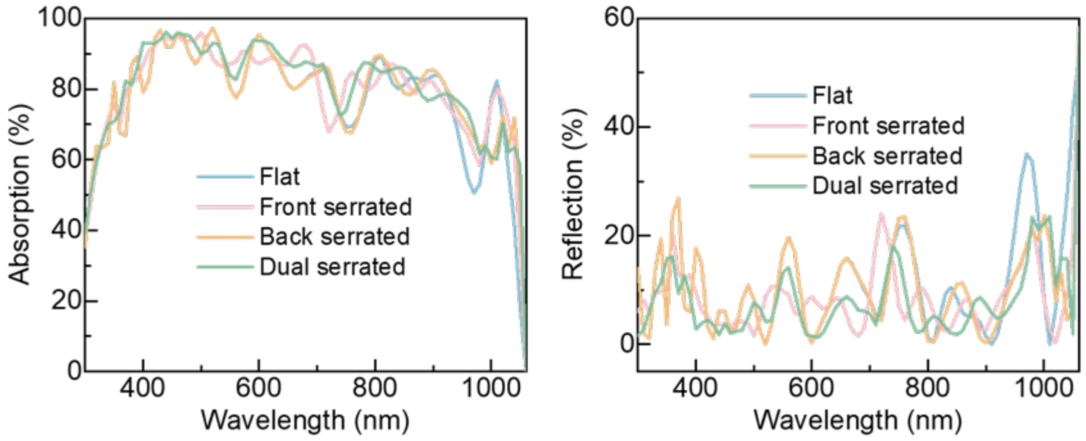

Fig. S3 a Absorption of the all-perovskite TSCs with four optimized structures. **b** Reflection of the all-perovskite TSCs with four optimized structures.

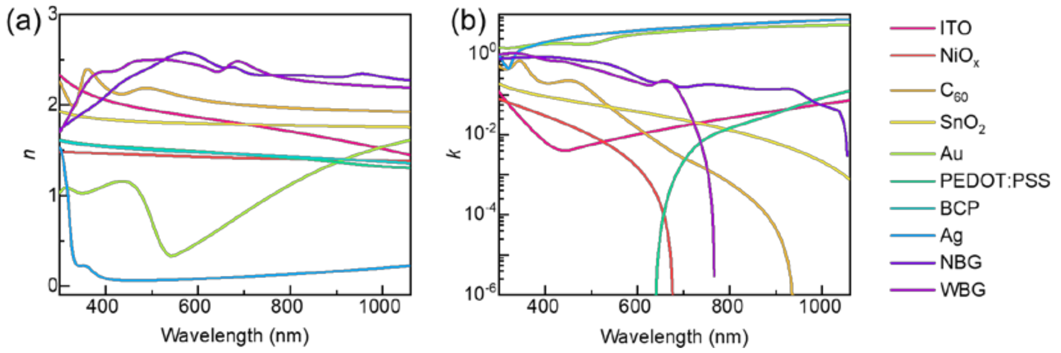

Fig. S4 **a** refractive index $n(\lambda)$ of each layer in the all-perovskite TSCs. **b** refractive index $k(\lambda)$ of each layer in the all-perovskite TSCs.

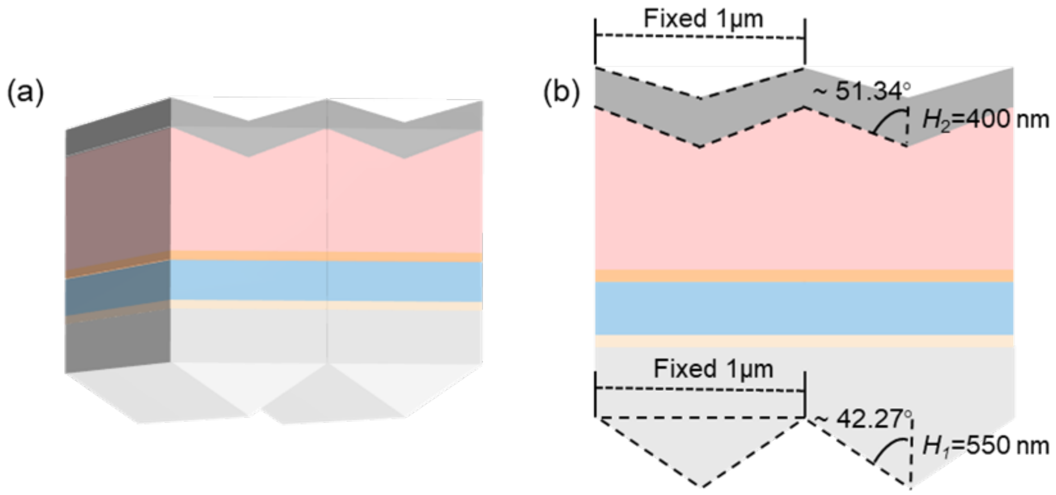

Fig. S5 **a** 3D schematic diagram of periodic dual serrated structure in the all-perovskite TSCs. **b** Widths, heights and angles of the optimized dual serrated structure.

Layer name	Thickness (nm)
Glass	1100000
ITO	180
NiO_X	7
1.8eV WBG PVSK ($DMA_{0.1}Cs_{0.4}FA_{0.5}Pb(I_{0.75}Br_{0.25})_{2.85}Cl_{0.15}$)	400
C_{60}	20
SnO_2	15
Au	1
PEDOT:PSS	18
1.25eV NBG PVSK ($Cs_{0.1}FA_{0.6}MA_{0.3}Sn_{0.5}Pb_{0.5}I_3$)	1200
C_{60}	20
BCP	5
Ag	150

Table S1 The materials and corresponding thicknesses used in the flat structure of the all-perovskite tandem solar cells.

Year	Photocurrent density of WBG subcell (mA cm^{-2})	Photocurrent density of NBG subcell (mA cm^{-2})	Ref.
2024	16.60	16.60	[1]
2025	16.22	16.21	[2]
2025	16.34	16.53	[3]
2025	16.23	15.96	[4]
2025	16.01	15.96	[5]
**2025**	**16.42**	**16.50**	**This work**

Table S2 The integrated photocurrent density of subcells from EQE measurements in recent years.

References

1. Wang, Y., Lin, R., Liu, C., Wang, X., Chosy, C., Haruta, Y., Bui, A. D., Li, M., Sun, H., Zheng, X., Luo, H., Wu, P., Gao, H., Sun, W., Nie, Y., Zhu, H., Zhou, K., Nguyen, H. T., Luo, X., Li, L., Xiao, C., Saidaminov, M. I., Stranks, S. D., Zhang, L. & Tan, H.: Homogenized contact in all-perovskite tandems using tailored 2D perovskite. Nature **635**(8040), 867-873 (2024).

2. Song, Z., Sun, K., Meng, Y., Zhu, Z., Wang, Y., Zhang, W., Bai, Y., Lu, X., Tian, R., Liu, C. & Ge, Z.: Universal approach for managing iodine migration in inverted single-junction and tandem perovskite solar cells. Adv. Mater. **37**(3), e2410779 (2025).

3. Liu, Z., Lin, R., Wei, M., Yin, M., Wu, P., Li, M., Li, L., Wang, Y., Chen, G., Carnevali, V., Agosta, L., Slama, V., Lempesis, N., Wang, Z., Wang, M., Deng, Y., Luo, H., Gao, H., Rothlisberger, U., Zakeeruddin, S. M., Luo, X., Liu, Y., Gratzel, M. & Tan, H.: All-perovskite tandem solar cells achieving >29% efficiency with improved (100) orientation in wide-bandgap perovskites. Nature Materials **24**(2), 252-259 (2025).

4. Ge, Y., Zheng, L., Wang, H., Gao, J., Yao, F., Wang, C., Li, G., Guan, H., Wang, S., Cui, H., Ye, F., Shao, W., Zheng, Z., Yu, Z., Wang, J., Xu, Z., Dai, C., Ma, Y., Yang, Y., Guan, Z., Liu, Y., Wang, J., Lin, Q., Li, Z., Li, X., Ke, W., Grätzel, M.

& Fang, G.: Suppressing wide-angle light loss and non-radiative recombination for efficient perovskite solar cells. Nature Photonics **19**(2), 170-177 (2025).

5. Yang, X., Ma, T., Hu, H., Ye, W., Li, X., Li, M., Zhang, A., Ge, C., Sun, X., Zhu, Y., Yan, S., Yan, J., Zhou, Y., Li, Z. a., Chen, C., Song, H. & Tang, J.: Understanding and manipulating the crystallization of Sn–Pb perovskites for efficient all-perovskite tandem solar cells. Nature Photonics (2025).
